# Anti-Proliferation and Anti-Invasion Effects of Diosgenin on Gastric Cancer BGC-823 Cells with HIF-1α shRNAs

**DOI:** 10.3390/ijms13056521

**Published:** 2012-05-24

**Authors:** Zhu-Jun Mao, Qian-Jue Tang, Ci-An Zhang, Zhi-Feng Qin, Bin Pang, Pin-kang Wei, Bo Liu, Yuan-Neng Chou

**Affiliations:** 1 Department of Obstetrics and Gynecology, Longhua Hospital Shanghai University of Traditional Chinese medicine, Shanghai 200032, China; E-Mails: zhujunlina@hotmail.com (Z.-J.M.); quinjue75@sohu.com (Q.-J.T.); 2 Department of Traditional Chinese medicine, Shanghai Changzheng Hospital, Senond Military Medical University, Shanghai 200003, China; E-Mails: happydrtcm@hotmail.com (C.-A.Z.); qinzf@126.com (Z.-F.Q.); 3 Air Force Center of Aviation Medical Evaluation and Training, Dujiangyan 611833, China; E-Mail: pangbin88@yahoo.com.cn; 4 Cardiovascular Department, Shanghai ChangHai Hospital, Second Military Medical University, Shanghai 200003, China; 5 Shanghai University of Traditional Chinese medicine, Shanghai 201203, China; E-Mail: john_chou21656@hotmail.com

**Keywords:** gastric cancer, HIF-1α, hypoxia, shRNA, diosgenin

## Abstract

Drug resistance is a major factor for the limited efficacy of chemotherapy in gastric cancer treatment. Hypoxia-inducible factor-1α (HIF-1α), a central transcriptional factor in hypoxia, is suggested to participate in the resistance. Here, we identified a hypoxia-mimic (cobalt chloride) sensitive gastric cell line BGC-823 to explore whether diosgenin, an aglycone of steroidal saponins, can inhibit cancer cell invasion and survival of solid tumor in a hypoxic mimic microenvironment. We have shown that diosgenin is a potent candidate for decreasing the ability of invasion and survival in cobalt chloride treated BGC-823 cells. In addition, when combined with HIF-1α specific short hairpin RNA (shRNA), diosgenin can inhibit BGC-823 cells more effectively. The anti-invasion role of diosgenin may be related to E-cadherin, integrinα5 and integrin β6. These results suggest that diosgenin may be a useful compound in controlling gastric cancer cells in hypoxia condition, especially when combined with down-regulated HIF-1α.

## 1. Introduction

Drug resistance is a major cause of the limited efficacy of chemotherapy in the majority of gastrointestinal malignancies, including gastric cancer [1,2]. It is known that the inherent specific genetic background of the tumor cell mutations, and epigenetic alterations after antiproliferative therapy can result in drug resistance [3,4]. Hypoxia, acidosis, and inflammation related to tumor microenvironment may be the main contributors for the drug resistance. Hypoxia is thought to be a hallmark of solid tumors and associated with metastases, therapeutic resistance, and poor patient survival [5]. Hypoxia-inducible factor-1 (HIF-1), a central transcriptional factor for cellular adaptation to hypoxia, has been implicated in drug resistance [6,7].

HIF-1 belongs to the basic helix-loop-helix family and requires the heterodimerization of HIF-1α and HIF-1β subunits for its activity [8]. Under normoxic conditions, HIF-1α protein is negatively regulated by proteasomal degradation such that intracellular levels of HIF-1α are almost undetectable [9]. The exposure to hypoxia or hypoxia-mimetic compounds, HIF-1α and consequently HIF-1 binding activity are dramatically increased in various cell lines [10].

HIF-1α is centrally involved in multiple aspects of tumorigenesis including tumor angiogenesis, proliferation, metabolism, metastasis, differentiation, as well as responses to radiation and chemotherapy [11]. The expression of HIF-1α is commonly increased in a variety of human solid tumors and elevated HIF-1α expression is associated with poor patient outcome in pancreatic, glioblastoma, gastric carcinomas, *etc.* [11,12]. Furthermore, the contribution of HIF-1α to chemoresistance has been observed in several solid tumors, including gastric cancer [13,14]. Interestingly, inhibition of HIF-1α via RNA interference (RNAi) or pharmacological compounds has improved the anti-tumor efficacy in murine cancer models [15].

Diosgenin is an aglycone of steroidal saponins, which is found in several plants, including Dioscorea species, fenugreek, and Costus speciosus [16]. Extracts from these plants have been traditionally used to treat diabetes [17], hypercholesterolemia [18], and gastrointestinal ailments [19]. It is a principal raw material for the synthesis of hormonal products such as dehydroepiandrosterone, which is obtained after hydrolysis of steroidal saponins [20]. Diosgenin exhibits anti-proliferative and pro-apoptotic activities on cancer cells in vitro widely [21,22], including breast cancer [23], colorectal cancer [24], osteosarcoma [25], and leukemia. However, whether diosgenin could play anti-invasion and pro-apoptotic roles on gastric cancer cells has not been explored.

Here we show for the first time that diosgenin exerted a certain anti-invasion role on BGC-823 cells, which are sensitive to the hypoxia stimulation, and down-regulated HIF-1α could significantly improve its effects. These findings suggest that combined with decreased HIF-1α, diosgenin could be considered as a candidate to improve clinical therapy of gastric cancer.

## 2. Results and Discussion

### 2.1. Identification of a Hypoxia-Mimetic Chemical Sensitive Gastric Cancer Cell Line

**Figure 1:**
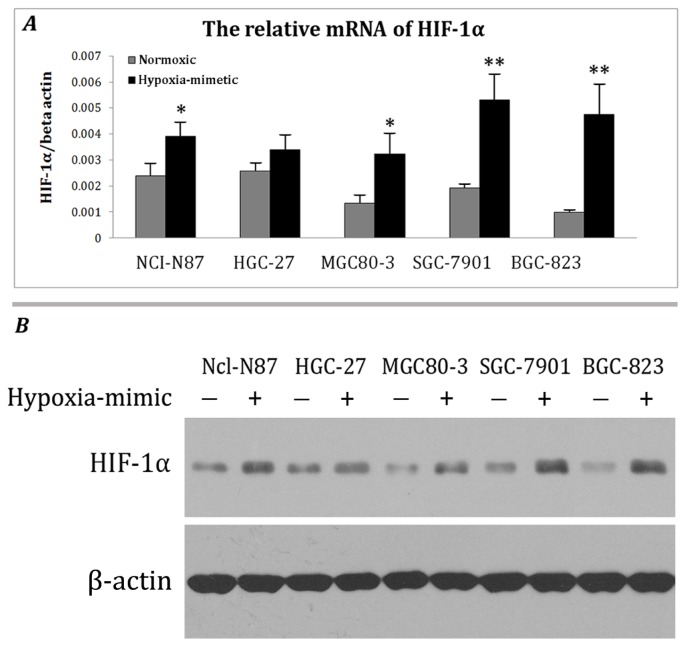
Identification of hypoxia mimetic chemical sensitive gastric cancer cell line. (**A**) Different cell lines were treated with 100 μM cobalt chloride and real-time PCR was performed to analyze the mRNA levels of HIF1α. The columns represent the gene expression of HIF1α compared to beta-actin, compared to the corresponding normal one. * *p* < 0.05 and ** *p* < 0.01; (**B**) Different cell lines were treated with 100 μM cobalt chloride for 24 hours and cell lysates were subjected to western blotting. β-actin served as the loading control. The results shown are representative of three independent experiments.

To identify the suitable gastric cell line, several cell lines were subjected to cobalt chloride treatment (a hypoxia mimic). The real-time PCR and Western blotting showed that cobalt chloride treatment increased HIF-1α expression in NCI-N87, MGC80-3, SGC-7901 and BGC-823 cells significantly (Figure 1). Compared to the other cell lines, the HIF-1α expression in BGC-823 was the lowest, but the increased effect of the hypoxia mimetic agent was the most significant. Therefore, BGC-823 cells were employed for the following experiments.

### 2.2. Screening of Effective shRNA Vector

**Figure 2:**
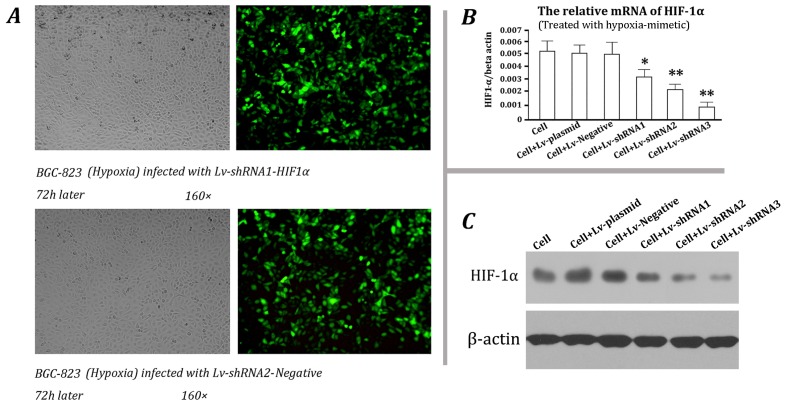
Selection of effective shRNA targeting HIF-1α. (**A**) BGC-823 cells were infected with (1) Lv-shRNA1-HIF1α and (2) Lv-shRNA1-Negative lentivirus, respectively, and the infection efficiency after 72 h was detected by microfluorography; (**B**) Total RNA was extracted from the infected cells or control cells which were treated with 100 μM cobalt chloride for 24 h and analyzed by real-time PCR. The expression of HIF1α mRNA was normalized by β-actin mRNA. * *p* < 0.05, ** *p* < 0.01 compared to the control; (**C**) Total proteins were extracted from the infected cells or control cells which were treated with 100 μM cobalt chloride for 24 h and analyzed by western blotting. β-actin served as the loading control. The results shown are representative of three independent experiments.

To inhibit HIF-1α stably, three candidate vectors expressing shRNAs targeting various regions of HIF-1α mRNA were constructed. Lentiviral particles were packaged and subsequently used for the infection of BGC-823 cells. After cobalt chloride treatment, the HIF-1α expression was analyzed. Fluorescence microscopy showed that the infection efficiency was higher than 90% (Figure 2a). At mRNA level, the negative lentivirus did not affect HIF-1α, but the three lentiviruses expressing shRNAs targeting HIF-1α can inhibit its expression significantly (*p* < 0.05 or *p* < 0.01, Figure 2b). The Western blotting results demonstrated the same trend, and the inhibitory efficiency of No. 3 lentivirus was the best (Figure 2c). Based on these results, it was selected for stable BGC-823 cell transduction.

### 2.3. HIF-1α Knockdown Enhances the Anti-Proliferation and Anti-Invasion Ability of Diosgenin

**Figure 3:**
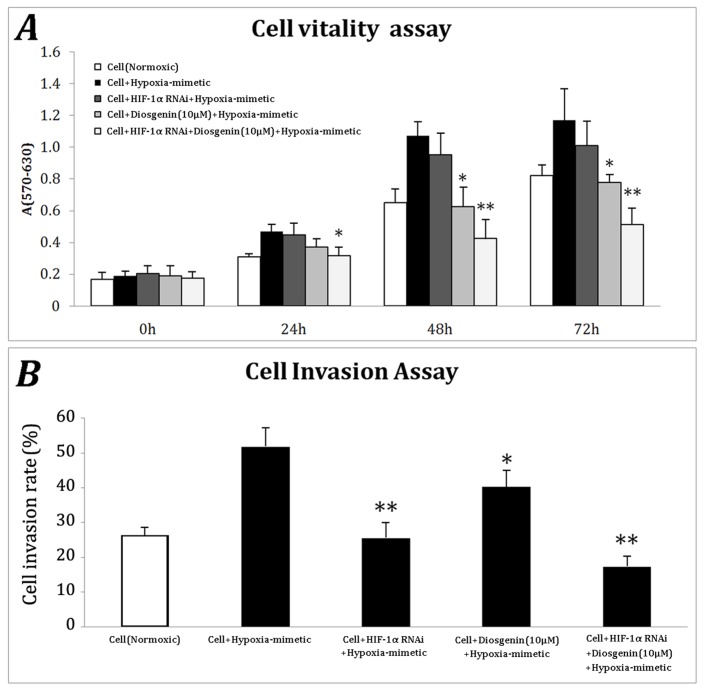
HIF-1α shRNA and diosgenin inhibit the enhanced proliferation and invasion of BGC-823 cells. (**A**) Normal BGC-823 cells and HIF1α-knockdown BGC-823 cells were seeded at 1 × 10^5^ cells/well on a 96-well plate. The hypoxia mimetic group was treated with 100 μM cobalt chloride with or without 10 μM diosgenin for the indicated hours. MTT assay was performed to assess the cell viability. * *p* < 0.05, ** *p* < 0.01 compared to the hypoxia mimetic group; (**B**) Cell invasion was analyzed using the QCM™ 24-well Invasion Assay Kit. 2.5 × 10^5^ cells were seeded into the upper chamber, and 0.5 mL RPMI 1640 medium with 10% FBS was added to the lower chamber. The hypoxia mimetic group was treated with 100 μM cobalt chloride with or without 10 μM diosgenin for 48 h. The invasive cells were detected using the reagents supplied in the kit. * *p* < 0.05, ** *p* < 0.01 compared to the hypoxia mimetic group.

To assess the anti-proliferation activity of diosgenin combined with HIF-1α silencing, the stable BGC-823 cells with HIF-1α shRNA and control cells were treated with cobalt chloride with or without diosgenin. The cell vitality was studied using MTT assay in the following 3 days of treatment. The hypoxia mimic increased the proliferation of control cells, and HIF-1α shRNA and diosgenin impaired this effect (Figure 3a). Moreover, the combination of HIF-1α knockdown and diosgenin treatment eliminated the rise caused by cobalt chloride treatment, and the differences between the group and cobalt chloride treated control culture were significant (*p* < 0.05 at 24 h, and *p* < 0.01 at 48 and 72 h). In addition, the Boyden transwell invasion model was employed to investigate the effect on cell invasion of the combination of HIF-1α silencing and diosgenin. We found that hypoxia mimetic chemical treatment can promote cell invasion and knockdown of HIF-1α could significantly inhibit the invasion, suggesting HIF-1α plays an important role in mediating cancer cells invasion under cobalt chloride treatment (Figure 3b). At the same time, diosgenin inhibited cobalt chloride-induced invasion less potently than HIF-1α knockdown. To our surprise, the combination of HIF-1α knockdown and diosgenin inhibited cell invasion was more significant than either treatment alone (Figure 3b). These findings implicated that HIF-1α mediates BGC-823 cell invasion and the anti-invasion and pro-apoptotic ability of diosgenin could be significantly improved when combined with down-regulated HIF-1α.

### 2.4. Anti-Invasion Role of Diosgenin Involves the Alteration of Cell Adhesive Molecules Expression

**Figure 4:**
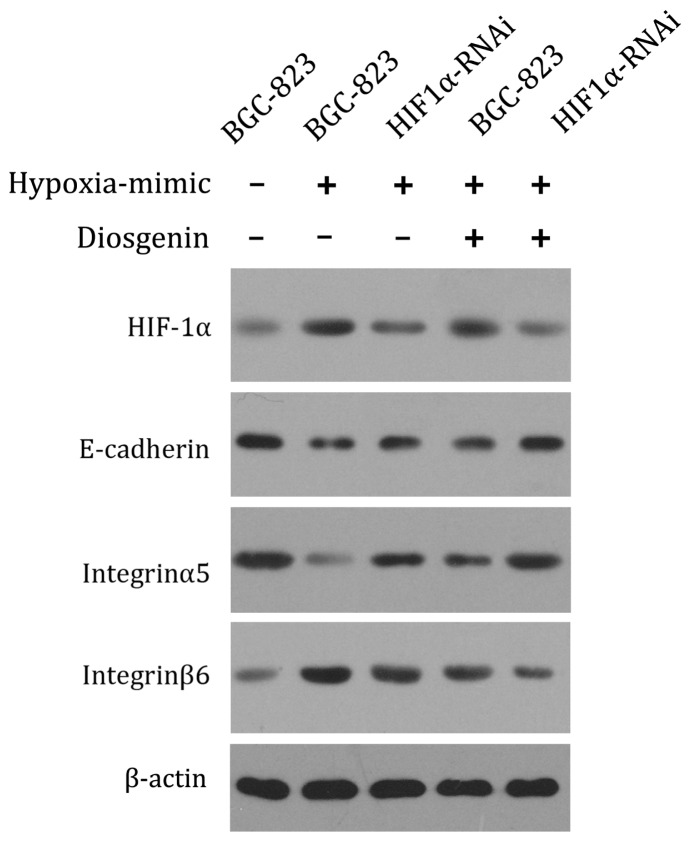
Western blot analysis of HIF1α, E-cadherin, integrinα5 and integrinβ6 protein expression in BGC-823 cells. Normal BGC-823 cells and HIF1α-knockdown BGC-823 cells were treated with or without 100 μM cobalt chloride, with or without 10 μM diosgenin and the total proteins were extracted and subjected to western blotting. β-actin served as the loading control. The results shown are representative of three independent experiments.

Since cancer cell invasion is related to adhesive molecules, to figure out whether the effects of HIF-1α and diosgenin are associated with these molecules, western blotting was used to analyze the changes of E-cadherin, integrinα5 and integrin β6. Our data showed that while cobalt chloride can down-regulate E-cadherin and integrinα5 expression, treatment of HIF-1α shRNA and/or diosgenin can significantly enhance their expression [26]. On the contrary, integrinβ6 showed the opposite change (Figure 4). These suggest the inhibitory effect on cell invasion of HIF-1α knockdown and diosgenin may involve the expression of E-cadherin, integrin α5 and integrin β6.

The reduced chemosensitivity of gastric cancer cells represents a pivotal obstacle in patient treatment. The transcriptional factor HIF-1α has been established as an important mediator of hypoxia-induced chemoresistance [27,28]. Here, we identify HIF-1α as a potential mediator of invasion and vitality of gastric cancer cells. Moreover, diosgenin can exert more potent anti-invasion and anti-proliferation roles when combined with down-regulated HIF-1α.

Tumor hypoxia plays important roles in mediating chemoresistance in cancer cells through which it impairs drug diffusion [29], reduces cell proliferation [30], decreases cytotoxic drug activity [31] and induces stress proteins [32]. Hypoxia also induces cellular adaptations, which contributes to cancer progression, such as initiation of angiogenesis and metastasis process, but also to tumor cell chemoresistance, one of these adaptations being the expression of multidrug resistance proteins such as ABC transporters [33]. While hypoxia has already been implicated in the resistance to chemotherapies by modifying gene expression [6], the exact mechanisms are not finely characterized.

As a primary transcription factor expressed in response to hypoxia, active HIF-1, a heterodimer consisting of α and β subunits, can be translocated into the nucleus and transcriptionally affect the expression of a number of genes through binding to hypoxia-responsive elements (HREs) [34]. Around 50 genes with HREs have been identified [29], including carbonic anhydrase IX (CA IX), glucose transporter 1 (Glut-1), erythropoietin (Epo), inducible nitric oxide synthase (iNOS), and vascular endothelial growth factor (VEGF). All of these may contribute to hypoxia related chemotherapeutical resistance and tumorigenesis.

In this study, we identified a gastric cancer cell line, BGC-823, which responded to the cobalt chloride treatment (hypoxia mimic) with more potential compared to the other examined gastric cell lines. Previous data showed that the inhibition of HIF-1α by means of RNA interference or pharmacological compounds exhibits antitumoral efficacy in murine cancer models [35]. Here, we employed lentiviral transduction of shRNA to inactivate HIF-1α and explored whether HIF-1α participates in hypoxia-related pro-invasion ability in gastric cancer cells. The results showed that HIF-1α knockdown can significantly inhibit cell invasion and decrease the cell vitality of BGC-823. It reminds us that HIF-1α could be a pivotal mediator in chemotherapeutical resistance in gastric cancer. This is confirmed by the latest finding that exposure to HIF-1α results in chemoresistance in gastric cancer cells through modulation of the p53 and NF-κB signaling pathway [36]. Therefore, the reduced expression of HIF-1α may impair the resistance of chemical therapy and be used in the combination therapy with chemotherapy drugs.

Diosgenin has been suggested to have potent anti-cancer effects [37]. In present studies, we showed that diosgenin could exert anti-invasion and anti-proliferation roles on gastric cancer cells with enhanced invasion and survival induced by hypoxia mimic. Moreover, it was surprisingly found that the combination of HIF-1α knockdown and diosgenin could play more potent roles than either of diosgenin or HIF-1α shRNA treatment alone. The results indicate that the anti-invasion effect of diosgenin on gastric cancer cells with cobalt chloride induced invasion and survival may be independent on a HIF-1α related signal pathway, so that the combination of diosgenin and down-regulated HIF-1α can produce synergetic effects. The mechanisms of how diosgenin modulates the proliferation and invasion in this model should be further elucidated.

The results of the experiments used to evaluate cell invasion in diosgenin treated BGC-823 cells with HIF-1α knockdown shows that the cell invasion was decreased. Therefore, cell adhesion molecules, including E-cadherin, integrin α5 and integrin β6, associated with the process were measured as well. Results show that combination treatment of diosgenin and HIF-1α silencing RNAs can enhance the expression of E-cadherin, an invasion/tumor suppressor gene [38], which is suggested to suppress the invasiveness of MDA-MB-231 and TSU-Pr1 cells in a tet-on inducible expression system [39]. Therefore, the enhanced E-cadherin may contribute to the anti-invasion effect of diosgenin and HIF-1α knockdown. In the context of tumor biology, the role of integrin α5β1 in tumor invasions has been revealed by the finding of lost or reduced integrin α5β1 expression in adenocarcinoma of the breast [40]. Diosgenin and HIF-1α silencing treatment enhanced integrin α5 expression in BGC-823 cells, which may partially account for the suppression of cell invasion and vitality. In addition, the combination treatment inhibited the expression of integrin β6, another cell adhesive molecule, which has proved to play a role in enhanced invasive behavior in oral squamous cell carcinoma [41]. Though we demonstrated that a few cell surface adhesion molecules are associated with the effects of combination of diosgenin and HIF-1α knockdown, more well-defined mechanisms are still to be determined.

## 3. Experimental Section

### 3.1. Cell Culture and Cobalt Chloride Treatment

Human gastric cell lines NCI-N87, HGC-27, MGC80-3, SGC-7901 and BGC-823 were obtained from Shanghai Cell bank of Chinese Academy of Sciences (Shanghai, China) and maintained in RPMI 1640 media (Invitrogen, USA) containing 10% fetal bovine serum (GIBCO-BRL) at 37 °C in a humidified atmosphere of 5% CO_2_. Twenty four hours after seeding, 10 mM of cobalt chloride solution was added to a final concentration of 100 μM and the cells were incubated for another 24 h. Then the relative HIF-1α mRNA and protein levels were analyzed. The cell line with a low expression of HIF-1α in normal conditions but the most significant increase when treated with the hypoxia mimic was selected for the following experiments.

### 3.2. Construction and Identification of Lentiviral Vectors for HIF-1α Silencing

Three shRNAs were selected based on the sequence of Homo sapiens hypoxia inducible factor 1 mRNA (HIF-1α, GenBank GI: 194473733) and a scrambled shRNA was used as a negative control. The target sequences and corresponding oligonucleotide sequences are shown in Table 1. The synthesized oligonucleotides (Invitrogen, China) were annealed and cloned into the pSIH1-H1-copGFP shRNA cloning and expression Vector (System Biosciences, USA). 293T cells (ATCC, USA) were transfected with the lentiviral vectors and two packaging plasmids using Lipofectamine™ 2000 (Invitrogen) following the manufacturer’s protocols and the viral supernatants were collected and used to infect the selected cells. 48 h after infection, the cells were treated with 100 μM cobalt chloride for 24 h. The relative HIF-1α mRNA and protein levels were analyzed. After the silencing efficiency was obtained, the optimal shRNA lentivirus was selected for stable transduction.

### 3.3. Cell Transduction, Stable Clone Selection, and Cobalt Chloride Treatment

The most effective lentivirus was used to infect the selected cells. The clonal stably-transduced cells with HIF-1α shRNA were obtained by limiting dilution and used for the subsequent experiments. The stable cells in the 3rd passage were subject to cobalt chloride treatment as described above. The relative protein levels of HIF-1α, E-cadherin, integrinα5 and integrinβ6 were analyzed by western blotting.

### 3.4. RNA Extraction and Quantitative RT-PCR

Cells were harvested in TRIzol Reagent (Invitrogen) and total RNA was isolated. The first-strand cDNA was synthesized using M-MLV Reverse Transcriptase (Takara) and random primers. Specific primers for quantitative PCR of human HIF-1α, E-cadherin, integrinα5, integrinβ6 and β-actin were as follows: HIF-1α: 5′-GCGCGAACGACAAGAAAAAGATAA-3′ and 5′-CACACGCAAATAGCTGAT GGTAAG-3′, E-cadherin: 5′-ACGGTAACCGATCAGAATGAC-3′ and 5′-GTCATTCTGATCGGTTA CCGT-3′, integrinα5: 5′-AGGAGCCTGTGGAGTACAAG-3′ and 5′-TGCTGCCCAGCTGAAATCTG AG-3′, integrinβ6: 5′-ATGAAGTTAACAGTGAAGAC-3′ and 5′-TTGCAAACACCATTTCCTCC AC-3′ and β-actin: 5′-CCTGTACGCCAACACAGTGC-3′ and 5′-ATACTCCTGCTTGCTGATCC-3′. Real-time PCR was performed using SYBR^®^ Premix Ex Taq™ kit (Takara, Japan) and TP800 System (Takara, Japan). cDNA from 100 ng total RNA was used as the template. The PCR amplification was carried out in the conditions: 40 cycles of denaturation at 95 °C for 10 s, annealing at 60 °C for 20 s and extension at 72 °C for 20 s. The mRNA levels of HIF-1α, E-cadherin, integrinα5, and integrinβ6 were normalized to β-actin using the ΔΔCt method.

### 3.5. Western Blotting

The total proteins were isolated with M-PER^®^ Mammalian protein extraction reagent (Pierce, USA). Protein concentration was determined by the BCA protein assay kit (Pierce, USA). The protein samples (10 μg) were separated by SDS–PAGE using a 12% (β-actin) or 10% (HIF-1α, E-cadherin, integrinα5, and integrinβ6) gel and electro-transferred onto PVDF membranes (Millipore, USA). The membranes were blocked with 5% non-fat milk in TBST and incubated with antibodies against HIF-1α (1:200), E-cadherin (1:300), integrinα5 (1:200), integrinβ6 (1:200) and β-actin (1:500) (Santa Cruz, USA) for 12 h at 4 °C. After washing, the membranes were then incubated with a HRP-labeled anti-goat or anti-mouse IgG antibody (Santa cruz, USA) and visualized by enhanced chemiluminescence reagents (Pierce, USA).

### 3.6. Cell Proliferation

The control and HIF-1α-knockdown cells were seeded into 96-well plates at 1 × 10^5^ cells/well 24 h before treatment. The cells were serum-starved for 8 h and then treated with or without 100 μM cobalt chloride and/or 10 μM diosgenin for indicated times. At the end, 10 μL MTT solution was added to each well. After incubation for additional 4 h, the violet crystals were dissolved in100 μL dimethyl sulfoxide (DMSO). The plates were vibrated at room temperature for 15 min and the absorbance of each well at 570 nm was measured with 630 nm as a reference.

### 3.7. Cell Invasion Assay

The Chemicon QCM™ 24-well Invasion Assay Kit was used to assess cell invasive potential following the manufacturer’s instructions. Briefly, the cells were harvested and resuspended with serum-free RPMI 1640 medium containing 5% bovine serum albumin at 1.0 × 10^6^ cells/mL. Approximately 0.25 mL cell suspension was added into the upper chamber. As a chemo-attractant, 0.5 mL RPMI 1640 medium with 10% FBS was added to the lower chamber. After 48 h of incubation, the invasive cells migrated through the polycarbonate membrane and attached to the lower surface were detached, and treated with lysis buffer/dye solution supplied by the kit. Aliquot mixes were transferred to a 96-well plate and read with Fluoroskan Ascent FL microplate fluorometer (Thermo Scientific) using 480/520 nm filter set. The Relative Fluorescence Unit (RFU) values correlated with the cell numbers were used to demonstrate invasion results, and the cell invasion rate was calculated by: cell invasion rate = the number of cell passing through the membrane/(cell number of upper chamber + number of cell passing through the membrane) × 100%. The experiments were performed three times in triplicate.

### 3.8. Statistical Analysis

Data were shown as the mean ± SD. Statistical analysis of the data was performed using Student’s *t*-test and *p* values < 0.05 were considered statistically significant.

## 4. Conclusions

In conclusion, we identified a hypoxia-mimic sensitive gastric cancer cell line BGC-823 and found that cobalt chloride can promote its ability to invade and survive. By knockdown of HIF-1α via lentiviral delivery of specific HIF-1α shRNA, the enhanced status of invasion and survival is effectively decreased. In addition, diosgenin, as a candidate contributing to new therapy approaches, is also explored since it can significantly inhibit the cobalt chloride induced invasion and survival in BCG823, especially when combined with down-regulation of HIF-1α. Subsequent studies should focus on whether diosgenin, when combined with shRNA of HIF-1α, is effectively applied to the animal gastric cancer model.

## Figures and Tables

**Table 1:** shRNA s targeting HIF-1α.

shRNA	Target sequence (5′–3′)	Oligonucleotide sequence(5′–3′)	
shRNA 1	GACTTTCCTCAGTCGACAC	Forward	GATCCGACTTTCCTCAGTCGACACCTTCCTGTCAGAGTGTCGACTGAGGAAAGTCTTTTTG
		Reverse	AATTCAAAAAGACTTTCCTCAGTCGACACTCTGACAGGAAGGTGTCGACTGAGGAAAGTCG
shRNA 2	GTCACCACAGGACAGTACA	Forward	GATCCGTCACCACAGGACAGTACACTTCCTGTCAGATGTACTGTCCTGTGGTGACTTTTTG
		Reverse	AATTCAAAAAGTCACCACAGGACAGTACATCTGACAGGAAGTGTACTGTCCTGTGGTGACG
shRNA 3	GTAGTGCTGACCCTGCACT	Forward	GATCCGTAGTGCTGACCCTGCACTCTTCCTGTCAGAAGTGCAGGGTCAGCACTACTTTTTG
		Reverse	AATTCAAAAAGTAGTGCTGACCCTGCACTTCTGACAGGAAGAGTGCAGGGTCAGCACTACG
shRNA-negative	GAAGCCAGATCCAGCTTCC	Forward	GATCCGAAGCCAGATCCAGCTTCCCTTCCTGTCAGAGGAAGCTGGATCTGGCTTCTTTTTG
		Reverse	AATTCAAAAAGAAGCCAGATCCAGCTTCCTCTGACAGGAAGGGAAGCTGGATCTGGCTTCG
